# Design and Selection of Engineered Lytic Proteins With *Staphylococcus aureus* Decolonizing Activity

**DOI:** 10.3389/fmicb.2021.723834

**Published:** 2021-09-14

**Authors:** Diana Gutiérrez, Lorena Rodríguez-Rubio, Patricia Ruas-Madiedo, Lucía Fernández, Ana Belén Campelo, Yves Briers, Martin Weiss Nielsen, Karl Pedersen, Rob Lavigne, Pilar García, Ana Rodríguez

**Affiliations:** ^1^Instituto de Productos Lácteos de Asturias (IPLA-CSIC), Asturias, Spain; ^2^Instituto de Investigación Sanitaria del Principado de Asturias, Oviedo, Spain; ^3^Laboratory of Applied Biotechnology, Department of Biotechnology, Ghent University, Ghent, Belgium; ^4^Laboratory of Gene Technology, Department of Biosystems, Katholieke Universiteit Leuven, Leuven, Belgium; ^5^Department of Microbiology and Production, National Food Institute, Technical University of Denmark, Lyngby, Denmark

**Keywords:** endolysin, protein engineering, antimicrobial, *Staphylococcus aureus*, skin decontamination

## Abstract

*Staphylococcus aureus* causes various infections in humans and animals, the skin being the principal reservoir of this pathogen. The widespread occurrence of methicillin-resistant *S. aureus* (MRSA) limits the elimination and treatment of this pathogen. Phage lytic proteins have been proven as efficient antimicrobials against *S. aureus*. Here, a set of 12 engineered proteins based on endolysins were conceptualized to select the most optimal following a stepwise funnel approach assessing parameters including turbidity reduction, minimum inhibitory concentration (MIC), time-kill curves, and antibiofilm assays, as well as testing their stability in a broad range of storage conditions (pH, temperature, and ionic strength). The engineered phage lysins LysRODIΔAmi and ClyRODI-H5 showed the highest specific lytic activity (5 to 50 times higher than the rest), exhibited a shelf-life up to 6 months and remained stable at temperatures up to 50°C and in a pH range from 3 to 9. LysRODIΔAmi showed the lower MIC values against all staphylococcal strains tested. Both proteins were able to kill 6 log units of the strain *S. aureus* Sa9 within 5 min and could remove preformed biofilms (76 and 65%, respectively). Moreover, LysRODIΔAmi could prevent biofilm formation at low protein concentrations (0.15–0.6 μM). Due to its enhanced antibiofilm properties, LysRODIΔAmi was selected to effectively remove *S. aureus* contamination in both intact and disrupted keratinocyte monolayers. Notably, this protein did not demonstrate any toxicity toward human keratinocytes, even at high concentrations (22.1 μM). Finally, a pig skin *ex vivo* model was used to evaluate treatment of artificially contaminated pig skin using LysRODIΔAmi (16.5 μg/cm^2^). Following an early reduction of *S. aureus*, a second dose of protein completely eradicated *S. aureus*. Overall, our results suggest that LysRODIΔAmi is a suitable candidate as antimicrobial agent to prevent and treat staphylococcal skin infections.

## Introduction

Methicillin-resistant *Staphylococcus aureus* (MRSA) is an important pathogenic bacterium for both humans and animals worldwide. Recent data shows that the percentage of invasive *S. aureus* isolates with resistance to methicillin ranges between 1.2 and 50.5% in European countries (EFSA and ECDC, 2017). Similarly, the values of MRSA strains exceed 20% in all WHO regions, and 80% in some countries (WHO, 2014).

The improvement of sanitation is an important strategy to prevent infectious diseases and limit the spread of resistant bacteria. Indeed, multiple areas of the human body can be colonized by *S. aureus*, including the nose as the most frequent carriage site, as well as the skin, perineum, and pharynx (Wertheim et al., 2005). Bacterial carriage is an important risk factor for nosocomial and surgical site infections (Wertheim et al., 2004; Wertheim and Vos, 2005). Therefore, nasal decolonization is recommended in patients undergoing cardiothoracic and orthopedic surgeries (Allegranzi et al., 2016). Recent data show that MRSA decolonization decreases the risk of surgical site infections by approximately 39% in total knee and hip arthroplasty surgery (Sadigursky et al., 2017). For prophylactic purposes, mupirocin has been usually prescribed as nasal ointment to remove *S. aureus* from the nose. However, recolonization by this bacterium was observed after treatment, mainly due to the selection of bacterial resistance to this antibiotic (Coates et al., 2009).

Prevention of the spread of *S. aureus* infection also relies on infection prevention and on the development of control intervention procedures (Rutala and Weber, 2019). Recently, prevention using a prophylactic vaccine has also been proposed (Moscoso et al., 2018). Still, epidemiological changes in MRSA have further complicated the development of a vaccine-based prevention strategy. For example, community-acquired methicillin-resistant *S. aureus* (CA-MRSA) has emerged as an important cause of MRSA infections replacing hospital-associated strains (HA-MRSA) in many countries from the Asia-Pacific region (Cho and Chung, 2017).

Bacteriophage-derived lytic proteins (endolysins and virion-associated peptidoglycan hydrolases) and their derived engineered proteins have been evaluated as efficient agents to remove or inhibit growth of undesirable bacteria in several areas (Schmelcher et al., 2012a; Rodríguez-Rubio et al., 2013). One of the most valuable characteristics of phage lytic proteins is the low probability to develop bacterial resistance, observed for different bacterial genera. This can be explained by the fact that peptidoglycan is an essential structure for bacterial viability (Fischetti, 2006).

Protein engineering based on the shuffling of the enzymatic modules of phage lytic proteins has been proven to be an efficient strategy to improve their lytic activity or stability compared to the natural proteins from where the domains are derived (Mao et al., 2013; Yang et al., 2014; Arroyo-Moreno et al., 2021; Lee et al., 2021), when used alone or in combination with antibiotics like oxacillin (Daniel et al., 2010) or bacteriocins like lysostaphin (Schmelcher et al., 2012b). Recently, a third generation of antimicrobials against *S. aureus* has been developed by immobilizing the engineered protein Auresine*Plus* (enzymatic activity domain from the staphylococcal autolysin LytM fused to the SH3b of lysostaphin) into a poly(lactide-co-glycolide)/chitosan matrix to be used as wound dressing (Urbanek et al., 2021).

Phage lytic proteins and their derived engineered proteins are highly active against *S. aureus*, also in animal models of infection, and several clinical trials with promising results are currently ongoing (reviewed by Gutiérrez et al., 2018). One of the potential applications of *S. aureus* endolysins is decolonization of the nares. In this context, several assays performed in mice and rats with pre-inoculated nares have resulted in the effective elimination of bacteria (Fenton et al., 2010; Paul et al., 2011; Yang et al., 2014; Imanishi et al., 2019; Wang et al., 2020). Looking at a future prophylactic application of endolysins for *S. aureus* skin decolonization (hospital patients, healthy workers, and food handlers), the efficacy of endolysins has been evaluated using *ex vivo* skin models (porcine and murine) (Pastagia et al., 2011; Fenton et al., 2013). The successful results of these experiments have subsequently led to clinical trials. Indeed, the lytic protein P128 (StaphTAME) is currently in phase 2 clinical trials for clearing nasal contamination in humans, particularly focusing on carrier patients with chronic dialysis treatments.^1^ Of note, one endolysin-based product (Staphefekt) intended for the treatment of human *S. aureus* skin infections (eczema, acne, and rosacea) is currently on the market, and its application is indicated to result in a clear improvement without generating bacterial resistance (Totté J. et al., 2017; Totté J. E. E. et al., 2017), although with unsuccessful results for treatment of atopic dermatitis (de Wit et al., 2019).

In our previous work, we identified and characterized two myophages phiIPLA-RODI and phiIPLA-C1C, which encode the endolysins LysRODI and LysC1C, respectively (Gutiérrez et al., 2015). These endolysins contain two catalytic domains (CHAP and amidase-2 domain) and one SH3b cell-wall binding domain (CBD). LysRODI showed an efficient *in vitro* activity against staphylococcal strains. Moreover, we have validated its activity in two *in vivo* models, a systemic infection model in zebrafish and a prophylactic assay against staphylococcal-induced murine mastitis (Gutiérrez et al., 2020). Another previously characterized endolysin, LysH5, also has two catalytic domains (CHAP and amidase-2 domain) and one SH3b CBD (Obeso et al., 2008). This endolysin shows lytic and antibiofilm activity against staphylococcal biofilm-producing strains (Gutiérrez et al., 2014). Likewise, we have recently characterized the endolysin LysA72, encoded by siphophage phiIPLA35, which belongs to the B2 morphotype (García et al., 2009b). This protein contains two catalytic domains (CHAP and amidase-3 domain) and one SH3_5 CBD and has antimicrobial activity, although it is mostly restricted to *S. aureus* strains (Gutiérrez et al., 2020).

This work aimed to design engineered lytic proteins from the endolysins LysH5, LysA72, LysRODI, and LysC1C, as well as the bacteriocin lysostaphin, by recombining their CHAP and binding domains in order to obtain better lytic, antibiofilm activities, resistance to hazardous environmental conditions, and prolonged shelf life. Among them, ClyRODI-H5 and LysRODIΔAmi stand out for their antimicrobial characteristics, the latter being selected for more in-depth *in vitro* and *ex vivo* studies.

## Materials and Methods

### Bacterial Strains and Culture Conditions

The staphylococcal strains (Table 1) used in this study were grown in tryptic soy broth (TSB; Difco, Franklin Lakes, NJ, United States) at 37°C with shaking (200 rpm) or on TSB plates containing 2% (w/v) bacteriological agar (TSA). *S. aureus* 15981 pSB2019 expressing GFP (Toledo-Arana et al., 2005) was grown on TSB media supplemented with 20 μg/ml of chloramphenicol. *Escherichia coli* DH10B (Invitrogen, Carlsbad, CA, United States) was used for cloning. *E. coli* BL21 (DE3) (EMD Biosciences, San Diego, CA, United States) was used for protein expression. *E. coli* strains were grown at 37°C with shaking in LB medium (1% tryptone, 0.5% yeast extract, and 1% NaCl) or on plates of LB supplemented with 2% (w/v) agar. For proper selection of the clones, 100 μg/ml of ampicillin (Sigma-Aldrich), 100 μg/ml of ampicillin supplemented with 5% sucrose (Sigma-Aldrich) or 50 μg/ml of kanamycin (Sigma-Aldrich) supplemented with 5% sucrose were used. Bacterial stocks were made adding 20% v/v glycerol to bacterial cultures and were kept at −80°C.

**TABLE 1:** Staphylococcal collection used in this work and MIC values of the engineered proteins.

Species	Strain	Origin	References	MIC (μM)											
				LysRODI ΔAmi	ClyRODI -Lyso	ClyRODI -H5	ClyA72 -RODI	ClyA72 -Lyso	ClyA72 -H5	ClyH5 -RODI	ClyH5 -Lyso	LysH5Δ Ami	ClyC1C- RODI	ClyC1C- Lyso	ClyC1C- H5
*S. aureus*	Sa9	Milk from cows with clinical mastitis	García et al. (2009a)	0.33	>14.54	1.84	>13.39	>19.32	>16.62	4.14	5.56	>25.57	4.24	7.58	>20.48
	15981	Human clinical isolate. Strong biofilm former	Valle et al. (2003)	0.17	>14.54	1.84	>13.39	>19.32	>16.62	4.14	5.56	>25.57	8.48	3.79	>20.48
	7829	Pig skin. MRSA	Unpublished	1.32	>14.54	1.84	>13.39	>19.32	>16.62	4.14	5.56	>25.57	8.48	7.58	>20.48
	2016-19142-2	Human clinical isolate. MRSA	Unpublished	0.66	>14.54	1.84	>13.39	>19.32	>16.62	4.14	5.56	>25.57	4.24	7.58	>20.48
	H1 9/3	Human clinical isolate. MRSA	Unpublished	0.66	>14.54	1.84	>13.39	>19.32	>16.62	4.14	5.56	>25.57	8.48	3.79	>20.48
	E10	Pig skin. MRSA	Unpublished	0.08	>14.54	1.84	>13.39	>19.32	>16.62	4.14	5.56	>25.57	8.48	7.58	>20.48
	Staph. 10	Human clinical isolate. MRSA	Unpublished	2.64	>14.54	1.84	>13.39	>19.32	>16.62	4.14	22.26	>25.57	8.48	7.58	>20.48
	Staph. 11	Human clinical isolate. MRSA	Unpublished	1.32	>14.54	1.84	>13.39	>19.32	>16.62	4.14	5.56	>25.57	8.48	7.58	>20.48
	Staph. 15	Human clinical isolate. MRSA	Unpublished	1.32	>14.54	1.84	>13.39	>19.32	>16.62	4.14	5.56	>25.57	4.24	7.58	>20.48
*S. epidermidis*	F12	Milk from woman with mastitis	Delgado et al. (2009)	2.64	3.63	7.37	>13.39	>19.32	>16.62	>33.12	>44.45	>25.57	4.24	15.16	>20.48
	B			2.64	>14.54	>14.76	>13.39	>19.32	>16.62	>33.12	>44.45	>25.57	4.24	15.16	>20.48
	DG2n			2.64	>14.54	>14.76	>13.39	>19.32	>16.62	>33.12	>44.45	>25.57	4.24	7.58	>20.48
	LO5081	Milk from healthy woman		1.32	>14.54	>14.76	>13.39	>19.32	>16.62	>33.12	11.11	3.19	4.24	15.16	>20.48
	LV5RB3			2.64	>14.54	>14.76	>13.39	>19.32	>16.62	>33.12	>44.45	>25.57	8.48	7.58	>20.48
*S. sciuri*	101	Milk from healthy woman	Martín et al. (2012)	2.64	>14.54	>14.76	>13.39	>19.32	>16.62	>33.12	>44.45	>25.57	8.48	7.58	>20.48
*S. hominis*	ZL31-13			1.32	>14.54	>14.76	>13.39	>19.32	>16.62	>33.12	>44.45	>25.57	8.48	3.79	>20.48
*Staphylococcus pasteuri*	ZL16-6			2.64	>14.54	>14.76	>13.39	>19.32	>16.62	8.28	2.78	>25.57	8.48	3.79	>20.48
*Staphylococcus xylosus*	ZL61-2			1.32	>14.54	>14.76	>13.39	>19.32	>16.62	2.70	5.56	>25.57	8.48	3.79	>20.48
*S. saprophyticus*	ZL112-15			0.33	>14.54	>14.76	>13.39	>19.32	>16.62	>33.12	22.26	>25.57	8.48	7.58	>20.48
*Staphylococcus arlettae*	ZL114-5			1.32	>14.54	>14.76	>13.39	>19.32	>16.62	1.04	5.56	>25.57	8.48	3.79	>20.48
*S. haemolyticus*	ZL89-3			0.66	3.63	3.68	6.69	>19.32	>16.62	8.28	2.78	>25.57	4.24	3.79	>20.48
*Staphylococcus gallinarum*	ZL90-5			1.32	1.82	>14.76	>13.39	>19.32	>16.62	>33.12	5.56	>25.57	8.48	3.79	>20.48
*Staphylococcus kloosii*	ZL74-2			1.32	>14.54	>14.76	>24.84	>15.24	>16.62	2.07	2.78	>25.57	8.48	7.58	>20.48

*MIC is expressed as the mode of three biological replicates.*

### Plasmid Construction and DNA Manipulation

The genes encoding LysRODI (Gene ID: 26623165), LysC1C (Gene ID: 26641066), LysH5 (Gene ID: 7057022), and LysA72 (Gene ID: 7057022) were optimized based on *E. coli* codon usage by the OptimumGene^TM^ codon optimization technology.^2^ Additionally, *Nde*I and *Xho*I restriction sites were added at the 5′- and 3′-end, respectively. The optimized sequences were synthetized and cloned into the pUC57 vector by GenScript (Township, NJ, United States). Then, genes were subcloned into the expression vector pET21a that introduces a C-terminal His_6_-tag and carries an ampicillin resistance gene. VersaTile technology was used for the construction of the engineered proteins (Gerstmans et al., 2020). First, VersaTile cloning was carried out to generate different tiles (each tile corresponds to a CHAP domain or an SH3 domain in this study; Table 2). To do this, a PCR reaction was performed adding subsequent *Bpi*I and *Bsa*I recognition and restriction sites at the 5′ and 3′ ends of the desired DNA fragment. Then, they were digested using *Bpi*I and introduced by ligation into the pVTEIII entry vector that harbors an ampicillin resistance gene and the negative sucrose selection marker (*sacB*). Finally, plasmids carrying the desired genes were transformed into competent cells of *E. coli* TOP10. All tiles were confirmed by Sanger sequencing and are stored at the VersaTile repository of Ghent University (Laboratory of Applied Biotechnology, Ghent University, Belgium). To obtain the engineered proteins, the desired tiles (for instance, CHAP domain from LysRODI and SH3 domain from LysH5) were digested with *Bsa*I and ligated into the expression vector pVTD3, which besides harboring the kanamycin resistance gene and the negative sucrose selection marker (*sacB*) also introduces a C-terminal His_6_-tag and a lactose promoter. All the plasmids carrying the parental endolysins and the DNA sequences coding for the different engineered proteins were transformed into *E. coli* BL21 (DE3) pLysS for protein expression.

**TABLE 2:** Principal characteristics and shelf-life storage of the engineered lytic proteins obtained in this work.

	EAD	CBD	Amino acids	MW(kDa)	Purification yield (mg/l)	Specific lytic activity (ΔOD × min^–1^ × μM^–1^)	MIC
LysRODI^*a*^	CHAP/Amidase_2	SH3	496	54.81	∼4.2	0.247 ± 0.003	3 months*
LysH5	CHAP/Amidase_2	SH3	481	53.7	∼1.6	0.028 ± 0.002	1 month
LysA72^*a*^	CHAP/Amidase_3	SH3	484	55.01	∼1.4	0.088 ± 0.003	1 week
LysC1C	CHAP/Amidase_2	SH3	484	55.01	∼2.6	0.008 ± 0.001	1 day
Lysostaphin	Endopeptidase	SH3	246	27	ND	0.029 ± 0.002	ND
LysRODIΔAmi (ClyRODI-RODI)	LysRODI CHAP	LysRODI SH3	287	31.56	∼3.6	0.645 ± 0.009	6 months*
ClyRODI-Lyso	LysRODI CHAP	Lysostaphin SH3	271	30.19	∼3.8	0.080 ± 0.002	1 week
ClyRODI-H5	LysRODI CHAP	LysH5 SH3	308	34.86	∼3.8	0.679 ± 0.008	6 months*
ClyA72-RODI	LysA72 CHAP	LysRODI SH3	276	30.77	∼2.2	0.122 ± 0.002	1 month
ClyA72-Lyso	LysA72 CHAP	Lysostaphin SH3	260	29.4	∼2.8	0.045 ± 0.000	1 week
ClyA72-H5	LysA72 CHAP	LysH5 SH3	297	34.08	∼1.6	0.019 ± 0.000	1 week
ClyH5-RODI	LysH5 CHAP	LysRODI SH3	282	31.07	∼3.2	0.048 ± 0.001	1 week
ClyH5-Lyso	LysH5 CHAP	Lysostaphin SH3	266	29.69	∼4.2	0.064 ± 0.007	1 month
LysH5DAmi (ClyH5-H5)	LysH5 CHAP	LysH5 SH3	303	34.37	∼2.8	0.013 ± 0.001	1 month
ClyC1C-RODI	LysC1C CHAP	LysRODI SH3	270	29.89	∼3.0	0.024 ± 0.002	1 week
ClyC1C-Lyso	LysC1C CHAP	Lysostaphin SH3	254	28.52	∼2.2	0.013 ± 0.001	1 week
ClyC1C-H5	LysC1C CHAP	LysH5 SH3	291	33.19	∼2.2	0.027 ± 0.002	1 day

The origin of catalytic and CBDs domains, molecular weight, purification yield, and specific lytic activity calculated against *S. aureus* Sa9 and expressed as ΔOD_600_ × min^–1^ × μM^–1^. Values represent mean ± SD of three biological replicates. Shelf-life storage was determined by keeping the purified proteins at 4°C and measuring activity after 1 day, 1 week, 1 month, 3 months, and 6 months. ND, not determined. ^a^Data already published in Gutiérrez et al. (2020). *Reduction of the specific lytic activity to half.

### Protein Overexpression and Purification

Protein expression was performed as previously described (Gutiérrez et al., 2020). Proteins were purified by immobilized metal ion affinity chromatography using 5 ml nickel-NTA Superflow resin columns (Qiagen, Valencia, CA, United States). Protein purity was evaluated in 12% (w/v) SDS-PAGE run at 150 V using Criterion precast gels (Bio-Rad, Inc., Hercules, CA, United States), and further revealed *via* conventional Coomassie staining.

Prior to any *in vitro* or *ex vivo* experiments the buffer was exchanged to 50 mM sodium phosphate (NaPi) buffer (pH 7.4) using the Kit “Zeba^TM^ Spin Desalting Columns, 7K MWCO, 5 ml” (Thermo Fisher Scientific, Madrid, Spain) following the supplier’s recommendations and finally filtered using 0.45 μm PES membrane filters (VWR, Spain). The final protein concentration was quantified using the Quick Start Bradford Protein assay (Bio-Rad, Hercules, CA, United States). In addition, protein LysRODIΔAmi was further purified by size exchange chromatography (SEC) prior to the *ex vivo* assays, at the company NANOVEX Biotechnologies S.L. (Asturias, Spain).

### Quantification of Specific Lytic Activity

Turbidity reduction assays were performed as previously described (Obeso et al., 2008) using *S. aureus* Sa9 cells suspended in 50 mM NaPi buffer (pH 7.4) and treated with twofold dilutions of the purified proteins. The OD_600__nm_ was measured at 37°C for 30 min. A 50 mM NaPi buffer alone was used as a control. Results were processed and expressed as the specific lytic activity (ΔOD_600_ × min^–1^ × μM^–1^) (Gutiérrez et al., 2020) using the Activity Calculator program (Briers et al., 2007). To measure the stability of the protein under prolonged shelf-life conditions, we kept the protein at 4°C and measured the specific lytic activity after 1 day, 1 week, 1, 3, and 6 months. In addition, to evaluate the effect of several cations on enzymatic activity, different salts (KCl, MgCl_2_, NaCl, MnCl_2_, ZnCl_2_, and CaCl_2_) were added at a final concentration of 1 mM to the turbidity assay buffer (50 mM NaPi buffer pH 7.4).

The stability of the proteins under different physicochemical conditions (temperature and pH) was evaluated by incubating an aliquot of the purified protein for 30 min in a wide temperature range (40, 50, 60, 70, 80, and 90°C). Then, the activity of the proteins at 37°C was determined as described above. In addition, the effect of pH on stability and activity was tested by diluting (1:100) the proteins into Britton–Robinson pH universal buffer (150 mM KCl, 10 mM KH_2_PO_4_, 10 mM sodium citrate, 10 mM H_3_BO_3_) adjusted to pH values ranging from 3 to 11. Finally, the activity of the proteins was measured by a classic turbidity reduction assay as described above but in the universal buffer adjusted at the different pH values. All experiments were performed using three independent biological replicates.

### Minimum Inhibitory Concentration

The minimum inhibitory concentration (MIC) of the proteins was determined in triplicate by the conventional broth microdilution technique in TSB (CLSI, 2015). In this assay twofold dilutions of each protein were added to a microtiter plate containing 10^5^ CFU/well living staphylococcal cells. The MIC was defined as the lowest protein concentration that inhibited visible bacterial growth after 24 h of incubation at 37°C. The final MIC values for each protein and strain correspond to the mode of three independent biological repeats.

### Time-Kill Assays

Exponentially growing cells (OD_600_ = 0.5) of *S. aureus* Sa9 in TSB medium were harvested by centrifugation (16,000 × *g*, 5 min), washed twice and suspended in 50 mM NaPi buffer (pH 7.4) to a final concentration of ∼10^7^ CFU/ml. A suspension containing 10^6^ CFU bacterial cells and 0.1 μM of each protein (5 ml) was incubated at 37°C with shaking at 200 rpm. At different time points (2–60 min), 50 μl were taken and 0.15 μg of proteinase K (Sigma-Aldrich, P2308; 30 U/mg) were added in order to stop the reaction. Appropriate dilutions of the suspensions were plated onto TSA and incubated at 37°C for 16 h. The antibacterial activity was quantified as the relative inactivation in log units [log_10_ (N_0_/N_i_) with N_0_ as the initial number of untreated cells and N_i_ as the number of residual cells counted after treatment]. All the experiments were performed using three independent biological replicates.

### Biofilm Assays

To assess the ability of proteins to prevent biofilm formation, *S. aureus* strain 15981 was grown as described previously (Gutiérrez et al., 2014) in the presence of different concentrations (0.002–2.5 μM) of each protein for 24 h at 37°C. Briefly, overnight *S. aureus* 15981 cultures were diluted in fresh TSBg [TSB supplemented with 0.25% w/v D-(+)-glucose] up to 10^6^ CFU/ml in TSBg containing the diluted protein. Then, 200 μl were poured into TC Microwell 96U w/lid nunclon D SI plates (Thermo Scientific, NUNC, Madrid, Spain), and incubated at 37°C for 24 h. The total biomass was determined after that time by crystal violet staining. The planktonic phase was removed and wells were washed twice with sterile 50 mM NaPi buffer (pH 7.4). The biofilm biomass adhered to the surface of the wells was determined by staining with crystal violet (0.1% w/v) for 15 min, followed by a gentle washing with water and de-staining in acetic acid (33% v/v). Absorbance was measured at a wavelength of 595 nm. All the assays were performed using three independent biological replicates.

Using this methodology, the minimal concentration of protein that prevented biofilm formation with more than 90% was established for the two most active proteins (0.15 μM). This value served as a baseline to treat 8 h preformed biofilms of *S. aureus* 15981, for which 1.5 μM (10× the above concentration) was used. The previously established protocol (Gutiérrez et al., 2014) was further followed for biofilm removal. Briefly, overnight *S. aureus* 15981 cultures were diluted in fresh TSBg (10^6^ CFU/ml), and 200 μl was poured into TC Microwell 96U w/lid nunclon D SI plates and incubated at 37°C for 8 h. Then, the planktonic phase was removed, the biofilm was washed twice with 50 mM NaPi buffer and the proteins added at a final concentration of 7 μM. Plates were incubated for 3 h at 37°C and the biomass measured as described above. In both assays (prevention and biofilm removal), 50 mM NaPi buffer was used as a blank and untreated cells of *S*. *aureus* 15981 as a control. All experiments were performed using three independent biological and three technical replicates.

### Cytotoxicity of LysRODIΔAmi Against a Keratinocyte Cell Line

The cytotoxicity of the protein was evaluated on a cell line of human keratinocytes HaCaT. The cell line was maintained under standard conditions in Dulbecco’s Modified Eagle’s Medium (DMEM)–high glucose (Sigma-Aldrich Co., St Louis, MO, United States) supplemented with 10% fetal bovine serum by incubating at 37°C and 5% CO_2_ atmosphere in a CO_2_-Series Shel-Lab incubator (Sheldon Manufacturing Inc., OR, United States) in 25 cm^2^ bottles with vented cap (Falcon^®^, Corning Inc. Life Science, Tewksbury, MA, United States) for 5 days. The cell monolayers were then washed in Dulbecco’s PBS Modified without CaCl_2_ and MgCl_2_ (Sigma-Aldrich, Madrid, Spain) and treated with Gibco^TM^ TrypLE^TM^ Express Enzyme (Thermo Fisher Scientific) for 20–30 min at 37°C, 5% CO_2_. Afterward, one volume of DMEM was added to stop the reaction. The cell suspension was centrifuged (300 × *g*, 5 min) to obtain the HaCaT pellet to be used at assays performed using the xCELLigence real-time cell analyzer (RTCA) (ACEA Bioscience Inc., San Diego, CA, United States).

The RTCA was used to detect any changes in the HaCaT cell line when exposed to different concentrations of highly purified LysRODIΔAmi. For this purpose, HaCaT cells were inoculated at a density of 2 × 10^4^ cells/well in 16-well E-Plates (ACEA Biosciences Inc.) containing 100 μl of DMEM per well, and then incubated at 37°C under a 5% CO_2_ atmosphere in a Heracell-240 Incubator (Thermo Electron LDD GmbH, Langenselbold, Germany) at 37°C under a 5% CO_2_ atmosphere. Approximately 20 h after seeding, the cells were treated with different protein concentrations (serial dilutions from 0.005 to 22.17 μM). The changes in the morphology and attachment/detachment of the cells upon the gold microelectrodes placed on the bottom of E-plates during exposure to LysRODIΔAmi were observed as impedance signal variations, recorded as Cell Index (CI units). After the incubation period the CI was expressed as Normalized CI at the 10-min time point after protein addition in order to calculate a dose-response curve. A decrease in the normalized CI was indicative of cytotoxicity. For each condition, measurements were carried out in triplicate.

### *Ex vivo* Skin Permeation Studies

The *ex vivo* permeation studies were carried out in a Franz Cell system (Hanson Research, Chatsworth, LA, United States) with a diffusion area of 1 cm^2^. The Franz Cell system was maintained at a constant temperature of 37°C by thermostatic bath circulation, while the receptor medium (0.9% NaCl) was stirred constantly at 350 rpm during the experiments. For the permeation studies, pig skin was purchased at Minimally Invasive Surgery Center *Jesús Usón* (Cáceres, Spain). Explants of 1 cm^2^ were prepared and carefully placed at the interface between the donor and receptor compartments. Aliquots (100 μl) of LysRODIΔAmi (15.84 μM), previously labeled with fluorescein using the kit Fluorescein-EX Protein Labeling Kit (Thermo Fisher Scientific, Madrid, Spain), were placed over the skin explants and samples were collected after 6 h. The skin was removed and the layers were separated with a surgical scalpel. The protein was extracted from the different layers and subsequently analyzed by fluorescence spectroscopy (excitation/emission 494/518 nm).

### *In vitro* Model of Wound Infection of HaCaT

To monitor the behavior of HaCaT cell monolayers upon exposure to *S. aureus* 15981 and further challenge with LysRODIΔAmi, we established an *in vitro* model of wound infection. The HaCaT monolayers were grown in 16-well E-plates as described above. Subsequently, the DMEM medium was removed and the cells were washed once with sterile phosphate-buffered saline (PBS; 137 mM NaCl, 2.7 mM KCl, 10 mM Na_2_HPO_4_, and 2 mM KH_2_PO_4_; pH 7.4). In selected wells, we introduced a mechanical wound with a thin sterile plastic tip by carefully scratching diagonally the monolayer grown on the bottom of the well. Other wells without wounds were also tested, as a model of a healthy HaCaT monolayer. Then, *S. aureus* 15981 was added at a final concentration of 10^7^ CFU/well, diluted in DMEM medium, and the plates were incubated for 3 h to allow bacterial adherence to the HaCaT cells and the initiation of biofilm formation. LysRODIΔAmi was further added to the wells at a final concentration of 0.7 μM. The plates were incubated for another 24 h and the CI was recorded. The results were expressed as normalized CI 10 min after adding the protein. Finally, the pH of the supernatant was measured after centrifugation of the 16-well plates, and the number of viable bacteria was determined by diluting and plating onto TSA after detaching the epithelial cell line. Each assay was performed in triplicate.

In addition, the effect of LysRODIΔAmi in the two *in vitro* models (healthy monolayer and wound infection monolayer) was visualized by confocal laser microscopy (CLSM). For this, the infection models were performed as described above but using eight-well μ-Slide with a glass bottom (ibiTreat, Ibidi GmbH, Germany) and *S. aureus* 15981 pSB2019 expressing GFP was grown supplementing the media with 20 μg/ml of chloramphenicol. At the end of the experiment (24 h treatment with LysRODIΔAmi), the supernatant of each well was removed and the monolayers were fixed with 1 volume (0.3 ml) of cold (−20°C) acetone for 10 min. Samples were washed twice with PBS for 5 min under mild stirring and permeabilized with PBS containing 0.1% Triton X-100 (Sigma-Aldrich, Madrid, Spain) for 15 min. The non-specific binding sites were blocked with fetal bovine serum (FBS) (diluted 25% in PBS) for 20 min, and finally washed once with PBS. The Phalloidin-Alexa-Fluor-568 probe (Molecular Probes-Thermo Fisher, Life Technologies S.A., Madrid, Spain) toward F-actin was added in 0.3 ml of PBS (final concentration 25 μL/ml) and samples were incubated overnight at 4°C in darkness. After washing twice with PBS, HaCaT nucleus were stained with DAPI (Merck-Millipore Cor., Billerica, MA, United States) used at 1:1000 (final dilution in PBS) and incubated under the same conditions for, at least, 6 h. Finally, samples were washed and 0.3 ml of PBS were added before being visualized under the microscope. For confocal scanning laser microscopy (CSLM), a Leica TCS AOBS SP8 X confocal microscope (Leica Microsystems GmbH, Heidelberg, Germany) was used. DAPI, Alexa-Fluor-568 fluorochromes and GFP were excited at 405 nm by a blue–violet laser diode and at 578 and 488 nm by a white light laser, respectively. Images were captured using the “Leica Application Suite X” software version 1.8.1.13759 (Leica).

### Pig Skin Model for Bacteria Decontamination

A version of the AOAC Germicidal Spray Products Test (official method 961.02) and a modified version of protocol established by Fenton et al. (2013), was used to test the ability of LysRODIΔAmi to eliminate *S. aureus* from pig skin. Briefly, pig skin purchased at the Minimally Invasive Surgery Center *Jesús Usón* (Cáceres, Spain) was carefully shaved with scissor and razor and cut into 2.5 × 2.5 cm explants. The skin was further cleaned with 70% of isopropyl alcohol, allowed to dry at room temperature for 30 min and put under UV light for 10 min. Sterile explants were then transferred to 24-well plates filled with 1 ml physiological saline solution (0.9% NaCl, pH 5.5) supplemented with 0.5% agar, to mimic human body conditions. Each explant was then inoculated with 10^5^ CFU/cm^2^ of *S. aureus* 15981 diluted in PBS. Bacteria were evenly distributed on the surface and allowed to adhere for 30 min at 37°C. Then, 200 μl of LysRODIΔAmi (2.6 μM/cm^2^; 16.5 μg/cm^2^) were sprayed onto the skin, whereas 200 μl of 50 mM NaPi buffer were sprayed for control purposes. The skin explants were subsequently incubated at 37°C for 24 h. In addition, a second treatment with the endolysin was carried out 8 h after the initial treatment. Samples were taken at each time point and 1.5 μL of proteinase K (Sigma-Aldrich, P2308-10MG; 30 U/mg) was added to inactivate the protein. For sampling purposes, two sterile cotton swabs were moistened in sterile PBS solution and used to sample each section of each skin piece by rotating and rubbing the swab, in a zigzag pattern, and repeating at right angles. The tips from each swab were placed into 5 ml of PBS and vigorously mixed for 1 min to dislodge cells using a vortex. Serial dilutions were made in PBS and colony forming units (CFUs) were determined by plating on TSA plates and incubated at 37°C for 16 h. The experiment was performed in triplicate.

### Statistical Analysis

The SPSS Statistics for Windows V. 22.0 (IBM Corp.) package was used to determine differences in activity between the lytic proteins. One-way analysis of variance (ANOVA) followed by the Student-Newman–Keuls *post hoc* test was used to determine differences among the activity of each protein at a level of significance of *p* < 0.05. On the other hand, Student’s *t*-test was performed to compare the differences between the treated and untreated bacterial cultures at a level of significance of *p* < 0.05. In most cases, data were expressed as the mean ± SD of three biological replicates.

## Results

### Design and Characterization of 12 Engineered Lytic Proteins Against *S. aureus*

Twelve new phage lytic proteins were designed by combination of catalytic and cell wall-binding (CBD) domains from the previously characterized endolysins LysH5 (53.7 kDa), LysA72 (53.2 kDa), LysRODI (54.5 kDa), LysC1C (53.2 kDa), and the bacteriocin lysostaphin (28.1 kDa). All engineered proteins have one CHAP catalytic domain and one SH3 CBD (Table 2). Coding sequences of each domain were cloned and combined using the VersaTile technology (Gerstmans et al., 2020). Following overexpression in *E. coli* BL21 (DE3) pLysS, all proteins were purified using nickel affinity chromatography with a purification yield ranging from 1.6 to 4.2 mg/L (Table 2).

To identify the most active engineered proteins, we followed a stepwise funnel approach. First, we performed an *in vitro* characterization of all variants (*n* = 12). To do that, we used turbidity reduction assays (including specific lytic activity measurements, stability analyses in terms of storage, resistance to elevated temperatures and non-physiological pH values, and analyses of the effect of ions) and MIC assays. Based on this pairwise comparison, we selected the most prominent candidates (*n* = 2) and analyzed their bactericidal activity with time-kill assays, as well as their *in vitro* capability to prevent biofilm formation and to eliminate preformed biofilms. Eventually, the best candidate was assessed for antibiofilm activity in an *ex vivo* model and we included the parental enzymes for comparison.

To start the identification of the optimal candidate, the specific lytic activity (ΔOD_600_ × min^–1^ × μM^–1^) of all 12 candidates against *S. aureus* Sa9 was determined in a turbidity reduction assay (Table 2). The four parental proteins were included. Two proteins showed the highest specific activity (LysRODIΔAmi and ClyRODI-H5), leading to an increase in activity of 2.6- and 2.7-fold compared to the parental protein LysRODI, and 22.8- and 24-fold compared with LysH5 (Table 2). Other proteins with higher activity compared to the parental proteins included ClyA72-RODI, ClyH5-RODI, and ClyH5-LYSO, displaying a 1. 4-, 1. 7-, and 2.2-fold higher activity than that of the parental protein donor of their catalytic domains, respectively. Analysis of the specific lytic activities after prolonged storage at 4°C (Table 2) indicated that LysRODIΔAmi and ClyRODI-H5 are the most stable over a 6-month period, retaining half of their specific lytic activity, whereas the LysRODI activity is retained for up to 3 months. This is in sharp contrast to two unstable proteins LysC1C and ClyC1C-H5 that lost their activity in just 1 day. The remaining proteins displayed stabilities between 1 week and 1 month.

To analyze protein stability of the engineered proteins under different physicochemical conditions, we first determined the effect of exposure to elevated temperatures within a range of 40–90°C on the specific lytic activity (Supplementary Figure 1A). While some lytic proteins (ClyRODI-LYSO and ClyH5-RODI) remained fully stable at 40°C, LysRODIΔAmi, ClyH5-LYSO, ClyC1C-RODI, and ClyC1C-LYSO retained 90, 64, 58 and 62% at 40°C, respectively. All remaining variants lost their lytic activity at 40°C. There is only one variant, ClyA72-H5 that retained approximately 40% of its activity at all the temperatures tested. For comparison, we previously reported that LysRODI has a fivefold decrease in activity after exposure to 40°C (Gutiérrez et al., 2020).

Regarding pH stability, most proteins were quite stable at pH values between 5 and 9, but their activity decreased (strongly) at pH 11. However, five proteins (LysRODIΔAmi, ClyRODI-LYSO, ClyRODI-H5, ClyA72-H5, ClyC1C-RODI, and ClyC1C-LYSO) stood out for their stability in the whole range of pH tested (pH values between 3 and 11) (Supplementary Figure 1B). In turn, seven engineered proteins (ClyRODI-H5, ClyA72-RODI, ClyH5-RODI, ClyH5-LYSO, LysH5DAmi, ClyC1C-RODI, and ClyC1C-LYSO) showed an increased activity after exposure to pH values between 3 and 5 (Supplementary Figure 1B). It is important to note that ClyC1C-H5 exhibited a twofold increase in activity after exposure to pH 5.

Finally, the influence of different ions (KCl, MgCl_2_, NaCl, MnCl_2_, ZnCl_2_, and CaCl_2_; 1 mM) on the lytic activity of the proteins was also evaluated (Supplementary Figure 1C). All lytic proteins were inactivated in the presence of ZnCl_2_ and, similarly, addition of MnCl_2_ also reduced the activity of some enzymes (Supplementary Figure 1C). Interestingly, CaCl_2_ resulted in an increased activity (up to fourfold) in the case of ClyH5-RODI. Finally, other ions (KCl, MgCl_2_, and NaCl) exerted little influence on the activity of the tested proteins. It is worth noticing the high sensitivity of protein ClyRODI-H5 to all the assayed ions (Supplementary Figure 2C).

The MIC of all 12 variants was determined against a panel of nine *S. aureus* strains (including MRSA) and 14 other isolates from ten different *Staphylococcus* species. The four parental endolysins were included as a reference (Table 1 and Supplementary Table 1). Among the engineered lysins LysRODIΔAmi and ClyRODI-H5 showed the lowest MIC values for the *S. aureus* strains tested. Comparing their MIC values to the MICs of their parental endolysins, LysRODI and LysH5, it is worth noting that LysRODIΔAmi is active against all the assayed strains and its MIC values (ranging from 0.08 to 2.64 μM) were lower than those of LysRODI for many strains belonging to *S. aureus*, *Staphylococcus epidermidis* and *Staphylococcus sciuri*. The highest reductions were observed for *S. aureus* 15981 and E10 with a sevenfold and 15-fold lower MIC, respectively. Regarding ClyRODI-H5, its MIC values were higher than those obtained for LysRODI against all strains but similar to or lower than the corresponding LysH5 MIC values. We selected a cut-off MIC of 2 μM against *S. aureus* strains, based on previously characterized proteins that were used for different *in vitro* and *in vivo* experiments with concentrations lower than 2 μM (reviewed by Gutiérrez et al., 2018), retaining LysRODIΔAmi and ClyRODI-H5 for further experiments.

### LysRODIΔAmi Shows Enhanced Bactericidal and Antibiofilm Activity

**FIGURE 1:**
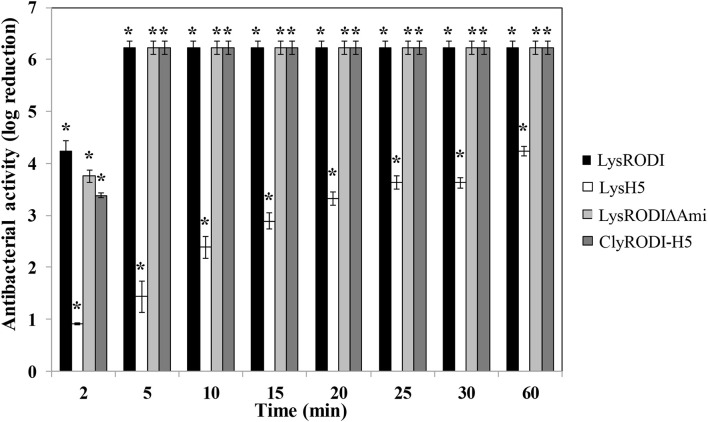
Time-kill curve of *S. aureus* Sa9 (10^6^ CFU/ml) treated with equimolar amounts of different engineered and parental proteins (0.1 μM). The results (means ± SDs of three replicates) are reported as bacterial reduction (log_10_ units) quantified as the relative inactivation in log units [log_10_(N_0_/N_*i*_); N_0_ is the initial number of cells and N_*i*_ is the number of residual cells counted after treatment]. Bars having an asterisk are statistically different (*p* < 0.05) from the untreated control, according to the Student’s *t*-test.

We subsequently performed time-kill assays for LysRODIΔAmi and ClyRODI-H5, including LysRODI and LysH5 as references (Figure 1). In these assays, LysRODI, LysRODIΔAmi, and ClyRODI-H5 showed higher bactericidal activity than LysH5. After 2 min, bacterial counts were reduced by almost 4 log units and after 5 min the bacterial counts were below the detection limit (∼6 log units). By contrast, LysH5 clearly showed a slower activity with a maximum removal of 4.3 log units, while complete elimination of the bacteria was not achieved even after 1 h of incubation.

**FIGURE 2:**
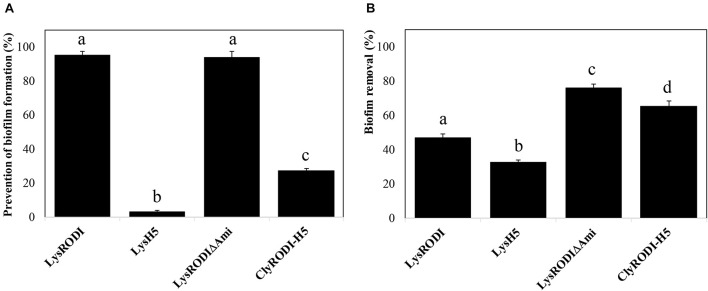
Antibiofilm activity of different engineered proteins against *S. aureus* 15981 biofilms. **(A)** Biofilm growth inhibition in the presence of 0.15 μM of the tested proteins. **(B)** Removal of 8-h *S. aureus* 15981 biofilms after addition of 1.5 μM of the tested proteins and further incubation at 37°C for 3 h. Bars represent the means ± SDs of six replicates. Bars having distinct lower case letters are statistically different (*p* < 0.05) from each other according to the ANOVA and SLK *post hoc* comparison test. The percentage of biofilm removal was calculated by crystal violet staining after the treatment.

To further assess the antibiofilm potential of the proteins, we first studied their ability to prevent biofilm formation on polystyrene at different concentrations. The proteins showed differences in antibiofilm activity (Supplementary Figure 2). In the case of LysRODI and LysRODIΔAmi, 0.15 μM was the minimum concentration that inhibited biofilm formation by more than 90%. In contrast, a much higher concentration (>1.25 μM) was necessary to observe a similar effect with ClyRODI-H5. Finally, endolysin LysH5 did not exhibit such antibiofilm activity at any of the concentrations tested, with a maximum decrease in biomass of 78% at 2.5 μM. In addition, it should be noted that, at lower concentrations, LysRODIΔAmi showed a fivefold higher prevention of biofilm formation compared to an equimolar amount (9 nM) of LysRODI. Similarly, when comparing the biofilm prevention ability of the different proteins at the same concentration (0.15 μM) (Figure 2A), we observed that LysRODI and LysRODIΔAmi completely inhibited biofilm formation, while ClyRODI-H5 and LysH5 only reduced biofilm development by 27 and 3%, respectively.

Subsequently, we analyzed the antibiofilm activity of the proteins against 8 h old mature biofilms (Figure 2B). LysRODIΔAmi and ClyRODI-H5 were the most efficient, respectively leading to 76 and 65.2% removal of preformed *S. aureus* 15981 biofilms. These values were at least 1.5-fold higher than those obtained with LysRODI and LysH5.

Integrating all data, LysRODIΔAmi was selected as the lead candidate from the 12 engineered variants. While its activity was equivalent to or slightly better than the activity of LysRODI against planktonic cells (MIC, time kill assay, and biofilm prevention), this protein excels in removal of established biofilm. In addition, its shelf-life is improved. Therefore, LysRODIΔAmi was further investigated for cytotoxicity and in decontamination experiments using *ex vivo* models.

### LysRODIΔAmi Prevents Biofilm Formation on a HaCaT Monolayer in a Wound Model Without Cytotoxic Activity

To develop a formulation based on a lytic protein for topical use, it is of key importance to determine its potential cytotoxicity. To examine this possibility, increasing concentrations of LysRODIΔAmi were added to cell cultures of the keratinocyte line HaCaT. Using the RTCA method, this protein did not display any toxicity to the cell line, as there was no reduction in cell viability, including at the highest concentration (22.17 μM) tested (Supplementary Figure 3). Of note, this concentration corresponds to 130 times the MIC of this lytic protein against *S. aureus* 15981.

**FIGURE 3:**
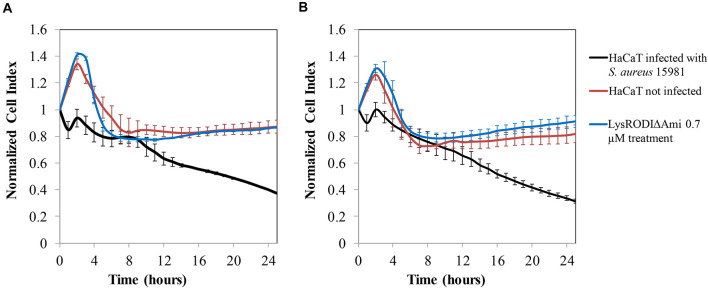
*In vitro* model of HaCaT cell line colonization by *S. aureus* 15981. **(A)** No wound damaged cell line; **(B)** wound damaged cell line. Variation in the normalized cell index (CI) of HaCaT monolayers contaminated with *S. aureus* 15981 (10^6^ CFU/well) for 3 h and treated with 0.7 μM of LysRODIΔAmi. Data normalization was performed as follows: the normalized-CI at a given time point was calculated by dividing the CI at this point by the CI at the normalization time point which, in our case, was the first time monitored immediately after protein addition (10 min). Thus, at the normalization time-point the normalized-CI equals 1 for all wells. The red, black, and blue lines represent the HaCaT cell line without bacteria, the infected cell line and the infected cell line treated with the protein, respectively. Values represent average ± SD of three replicates.

To assess the efficacy of LysRODIΔAmi to remove *S. aureus* bacteria *in vitro* from a biofilm grown on a HaCaT monolayer, a challenge study was performed using non-disrupted and disrupted (wound model) HaCaT monolayers. In the absence of the lytic protein, a decrease in the CI, indicative of monolayer destruction by the bacteria, was observed in both types of monolayers (Figure 3). In contrast, our results indicated that 0.7 μM LysRODIΔAmi was able to prevent degradation of the cell line due to bacterial growth (Figure 3). Besides, there were no remaining bacteria after the protein treatment. It is important to highlight that the results were similar for both non-disrupted and disrupted (wound model) HaCaT cells (Figures 3A,B, respectively). To confirm these results, we examined the morphology of the infected HaCaT cell line with or without treatment by CLSM and compared it to an uninfected keratinocyte culture (Supplementary Figure 4). Control cells (healthy HaCaT cell line) exhibited a normally stained nucleus and cytoplasm showing a typical F-actin cytoskeleton, thus having an epithelial-like morphology with well-connected annexed cells. In turn, bacterial-infected monolayers exhibited loss of the interconnection between F-actin filaments, and the nuclei are stained more intensely blue probably due to chromatin condensation (Supplementary Figure 4). In addition, green-stained bacterial cells can be observed between the keratinocytes, although not all bacteria in the experiment were expressing GFP. Conversely, the photographs obtained in infected monolayers after treatment with 0.7 μM LysRODIΔAmi were more similar to the control, and bacteria could not be observed. In the case of the wound model, we found the same effect, although there was no growth of the keratinocyte monolayer in the damaged area. Thus, we can conclude that LysRODIΔAmi activity allowed the maintenance of monolayer integrity, whereas the untreated samples showed degraded keratinocytes, which appeared deformed and without stained cytoplasm and nuclei, as a result of bacterial infection. A concentration- and time-dependent change in cell morphology and nuclear staining was observed. Cells exposed to the protein were alive after 24 h of treatment confirming that the protein is not cytotoxic for the cell line.

### LysRODIΔAmi Is Highly Effective for *S. aureus* Decontamination in *ex vivo* Assays

**FIGURE 4:**
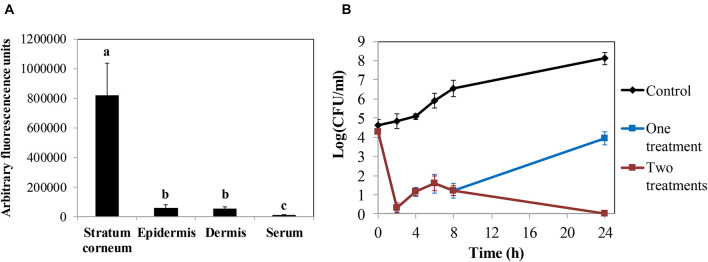
*Ex vivo* models in pig skin. **(A)** Penetration assay of LysRODIΔAmi measuring the concentration of protein (arbitrary fluorescence units) in every layer of a pig skin explant. Bars represent means ± SDs of three replicates. Bars having distinct lower case letters are statistically different (*p* < 0.05) according to the ANOVA and SLK *post hoc* comparison test. **(B)** Activity of LysRODIΔAmi against *S. aureus* 15981 in an *ex vivo* pig skin disinfection model. Data represent log(CFU/ml) for each explant after treatment with either a single dose of the lead variant (16.5 μg/cm^2^) or two doses of the protein (the second dose was administered 8 h after the first dose). The control represents the bacterial number without protein treatment. Values correspond to the mean ± SD of four replicates. The bacterial detection limit is 5 CFU per explant.

The promising results obtained in the keratinocyte cell line assays led us to test the efficacy of the protein in *ex vivo* decontamination experiments using pig skin. First, we established the ability of the protein to penetrate the skin layers in order to develop a possible topical application. We determined the ability of fluorescein-labeled LysRODIΔAmi to cross the skin layers using a Franz cell diffusion system in an *ex vivo* model of pig skin. Thus, measurement of the fluorescent signal allowed us to determine that LysRODIΔAmi was mostly located in the upper layer, the stratum corneum (820,866 ± 217,151 absorbance units), whereas only 57,287 ± 20,197 and 55,114 ± 10,114 absorbance units were detected in the epidermis and dermis, respectively (Figure 4A).

Finally, we performed an *ex vivo* model of skin artificially contaminated with *S. aureus* 15981 (10^5^ CFU/cm^2^) and sprayed with one dose of LysRODIΔAmi (16.5 μg/cm^2^), which resulted in almost complete removal of bacteria after 2 h (Figure 4B). However, after this initial bacterial reduction, a substantial bacterial regrowth was observed, reaching 3.9 log units at 24 h of incubation. However, this value is still lower than the viable counts detected on untreated samples, which reached values of 8.1 log units. In similar experiments, a second dose of the protein administered 8 h after the first treatment resulted in total bacterial removal after 24 h of incubation (Figure 4B).

## Discussion

Among the multiple clinical strategies used to prevent MRSA infections, universal decolonization (e.g., chlorhexidine gluconate baths and mupirocin) was shown to be cost-saving and prevented MRSA colonization of skin by 44% and MRSA infection by 45% (Gidengil et al., 2015). However, the development of bacterial resistance after repeated use of some decontamination compounds is an issue of major concern, and is of paramount importance in the search for alternative antimicrobials. Phage endolysins have evolved to bind a critical component of the bacterial cell wall that is difficult for the bacteria to change and, therefore, resistance has not been reported to date. This characteristic clearly makes them promising antimicrobial candidates. However, it is necessary to select lytic proteins with a high activity to counteract the economic cost of production of a commercial product. This selection process can be facilitated by taking advantage of their modular structure, which allows the combination of one or more enzymatically active domains connected by a short linker region with a specific cell wall-binding domain. In this work, we designed 12 novel chimeric anti-staphylococcal proteins derived from natural, previously characterized endolysins. These novel proteins contain one catalytic domain (CHAP) plus one CBD from the endolysins LysH5, LysA72, LysRODI, LysC1C, and the bacteriocin lysostaphin. We decided to use only the CHAP domain as previous reports had found that the amidase domain of staphylococcal phage endolysins is virtually inactive (Donovan et al., 2006).

According to previous reports, some chimeric proteins exhibit increased activity compared to their parental endolysins (Daniel et al., 2010; Mao et al., 2013; Rodríguez-Rubio et al., 2013; Arroyo-Moreno et al., 2021; Lee et al., 2021) or also show the ability to penetrate eukaryotic cells and kill intracellular bacteria, when fused to a cell penetrating peptide (Röhrig et al., 2020). Following a stepwise funnel approach combining turbidity reduction, MIC, time-kill, and biofilm assays, we eventually identified a leading candidate, LysRODIΔAmi, which contains the CHAP and CBD domains from LysRODI but lacks the amidase domain. This truncated protein showed high specific activity against *S. aureus* strains, exceeding LysRODI activity 2.6-fold. This result confirms previous observations that removal of the amidase domain results in an increase of their specific activity in some endolysins (Schmelcher et al., 2015). More recently, a role of staphylococcal amidase domains in enhancing the binding affinity of the CBD has also been proposed (Son et al., 2018), however, this role is apparently unnecessary for their use as antibacterial protein.

The chimeric protein ClyRODI-H5 was also very active against *S. aureus* and exceeded the specific lytic activity of LysRODI and LysH5 by 2.7-fold and 24-fold, respectively. This lytic protein contains the LysRODI CHAP domain fused to the LysH5 CBD domain. It is worth noting that the CHAP domain is the most abundant domain in staphylococcal peptidoglycan hydrolases, including endolysins (Oliveira et al., 2013). Generally, this domain displays increased activity when fused to CBDs such as the lysostaphin SH3b domain (Paul et al., 2011; Rodríguez-Rubio et al., 2012; Schmelcher et al., 2015; Röhrig et al., 2020). However, it does not seem to be a general rule, as some chimeric proteins (ClyRODI-LYSO, ClyA72-LYSO, ClyH5-LYSO, and ClyC1C-LYSO) were not more active than their parent proteins, even though all of them contain one CHAP domain fused to the lysostaphin SH3b domain.

The catalytic CHAP domain from LysRODI seems to be the most active among all the catalytic domains tested in this work. Indeed, engineered proteins containing this domain (LysRODIΔAmi, ClyRODI-LYSO, and ClyRODI-H5) showed the highest specific lytic activity. In contrast, those carrying the CHAP domain from LysC1C had the lowest specific lytic activity, which might be related to the fact that phage C1C preferably infects *S. epidermidis* over *S. aureus*. Concerning the CBD from LysRODI, designer proteins containing this domain (LysRODIΔAmi, ClyA72-RODI, ClyH5-RODI, and ClyC1C-RODI) also had a higher specific activity than their counterparts carrying domains from other proteins, with the exception of ClyH5-RODI which showed a slightly lower activity than ClyH5-LYSO.

One of the potential applications of lytic proteins is topical decontamination. With this in mind, we assessed the stability of the engineered proteins under non-physiological conditions that may occur during processing of the product or during application on the skin. As expected, all proteins turned out to be sensitive to high temperatures but three proteins (LysH5, ClyRODI-LYSO, and ClyH5-RODI) seem to be more stable, and retained their activity after incubation at 40°C for 30 min. Additionally, other three proteins (ClyH5-LYSO, ClyC1C-RODI, and ClyC1C-LYSO) displayed a twofold reduction in their enzymatic activity. Regarding pH, we were especially interested in examining the effect of acidic pH values, as the skin pH typically ranges between 4.5 and 5.9. In general, the proteins were active at pH <9, with a high activity reduction at pH 11. It is worth noting that most of the tested proteins exhibited increased lytic activity at pH values near 5, with the exception of LysA72, which was less active at this pH. Given that the sweat glands secrete different molecules, including water and electrolytes, onto the skin surface, it was important to confirm that the lytic activity of most engineered proteins was not modified in the presence of sodium chloride and potassium chloride.

Due to the high diversity of the skin microbiome, which consist of bacteria, fungi, and viruses and their important impact on human health (Schommer and Gallo, 2013), a broad bactericidal spectrum against bacteria is not recommended as a decontamination candidate. Among skin-colonizing bacteria, staphylococci are the most abundant, and some of them like *S. epidermidis* or *Staphylococcus hominis* are found in virtually all body parts. Others such as *Staphylococcus capitis* and *Staphylococcus auricularis* are only present at a specific age or in certain parts of the human body, respectively (Cheung et al., 2010). However, endolysins have a quite narrow specificity against their target bacteria, which would allow keeping the skin microbiota undisturbed after treatment. In this context, we investigated the bactericidal activity of several new proteins compared with their parental endolysins. To do that, we determined their MICs for *S. aureus*, *S. epidermidis* and nine strains from different staphylococcal species. LysRODIΔAmi showed the same pattern as LysRODI in terms of activity spectrum, which means that bactericidal activity was mainly observed for *S. aureus*, *S. epidermidis*, *Staphylococcus saprophyticus*, and *Staphylococcus haemolyticus*, all of them an important cause of infections. Regarding ClyRODI-H5, MIC values were slightly higher than those obtained for LysRODI but lower than those of LysH5 in all *S. aureus* strains. Similar to LysRODIΔAmi, ClyRODI-H5 proved to be less active against *S. epidermidis* strains than its parental proteins. Overall, it is worth highlighting that the LysRODIΔAmi showed similar MIC values to those previously reported for CF-301 against 103 methicillin-sensitive *S. aureus* (MSSA) and 120 MRSA strains (Schuch et al., 2014).

Environmental and local factors of the human skin also include host defense molecules, like proteases, that might degrade endolysins. In such a context, rapid killing would definitely be an asset. Analysis of the killing kinetics of LysRODIΔAmi and ClyRODI-H5 revealed a fast bactericidal activity against *S. aureus*, albeit slightly slower than that of the parental endolysin LysRODI. However, it must be noted that the three proteins had a similar killing rate at 5 min of incubation. The rapid removal of *S. aureus* cultures was also described for other endolysins such as HY-133, which was able to remove 10^6^ CFU/ml in 4 min by adding 16 × MIC (Knaack et al., 2019). Similarly, protein CF-301 reached bactericidal levels (≥3-log_10_ CFU reduction) at its minimum inhibitory concentration (1 × MIC) within 30 min (Schuch et al., 2014).

One reason for the extraordinary persistence of *S. aureus* in both biotic and abiotic surfaces is its ability to form biofilms, and its resistance to antimicrobials including decontamination agents. In this study, we demonstrated that LysRODIΔAmi can remove mature staphylococcal biofilms more efficiently than LysRODI and can also prevent the establishment of these structures on an abiotic surface. The disruption of staphylococcal biofilms by phage lysins has previously been reported by several authors (Sass and Bierbaum, 2007; Son et al., 2010; Jun et al., 2013; Gutiérrez et al., 2014, 2017; Melo et al., 2018). Beyond their antibiofilm activity, endolysins also display a synergistic effect when combined with extracellular-matrix degrading enzymes (Olsen et al., 2018), antibiotics (Chopra et al., 2015), or phages (Duarte et al., 2021). Moreover, subinhibitory doses of some endolysins can inhibit biofilm formation by *S. aureus* strains due to their ability to provoke the downregulation of several genes coding for bacterial autolysins that are known to participate in staphylococcal biofilm development (Fernández et al., 2017).

The skin is composed of several morphologically distinct layers, of which the outermost layer, the stratum corneum, is a protective barrier (Bouwstra and Ponec, 2006). Most drugs applied on the skin permeate across this layer. For this reason, we were interested in finding out how deep it is possible to deliver the endolysin through the skin, and to what extent it might be cytotoxic and interact with the immune system. In this context, we used LysRODIΔAmi in all subsequent experiments because it is the most active protein and has suitable characteristics in terms of its stability and antibiofilm activity, possibly due to its smaller size or a difference in overall surface charge which would enable deeper penetration into the layers of the biofilm. Our study confirmed that this engineered protein is only able to penetrate the stratum corneum and to slightly reach the epidermis and dermis. This observation allowed us to conclude that the protein is unlikely to penetrate the healthy skin, reducing the risk of an immune response. Previous studies revealed that some endolysins like LysGH15 and ClyS triggered the generation of specific antibodies in mice only when they were administered subcutaneously without inhibiting their lytic activity (Rashel et al., 2007; Pastagia et al., 2011; Gilmer et al., 2013; Zhang et al., 2016). This might be due to the fact that the generated antibodies do not block the binding and catalytic domains of the protein. In turn, it is also possible that the affinity of these lytic proteins for their bacterial target is higher than the antibody-endolysin binding affinity or that the action of lysins is quicker than neutralization by circulating antibodies. Moreover, antibody production was also observed when LysGH15 was used to treat wounded skin but the antibody titer was very low (Zhang et al., 2016). Also, endolysin LysEF-P10 enhanced the IgG levels but did not induce the production of IgM and IgE; therefore, it seems that the risk of an undesirable immune response derived from repeated administration of this protein is low (Cheng et al., 2017).

After demonstrating that LysRODIΔAmi did not exert any significant cytotoxic effect on human cells at concentrations of up to 22.1 μM, we examined its effect on the bacterial load, in an undamaged keratinocyte monolayer (HaCaT) and in a mechanical wound damage monolayer treated with the proteins. Clearly, an important factor in accelerating wound closure and wound healing is decolonization of the *S. aureus* strain. Actually, previous reports have shown the ability of phage endolysins to remove MRSA cells in a mouse model of skin wound infection and, in combination with apigenin and aquaphor, significantly accelerated wound healing (Cheng et al., 2018). In some cases, endolysins can be modified to improve their activity. In this regard, the endolysin JDlys was modified by fusion to a cell-penetrating peptide able to kill MRSA bacteria causing intracellular infections (Wang et al., 2018), which was also recently demonstrated, with reductions of more than 4.5 log units of intracellular bacterial killing when using chimeric lysins fused to cell penetrating peptides (Röhrig et al., 2020). Moreover, endolysins fused to cationic peptides (Artilysins) have shown a high activity in keratinocyte monolayers against infections caused by opportunistic pathogens such as *Pseudomonas aeruginosa* and *Acinetobacter baumannii* (Briers et al., 2014; De Maesschalck et al., 2020).

Finally, the present study also demonstrated the potential of LysRODIΔAmi for removing *S. aureus* from the surface of pig skin. When it was applied on previously contaminated skin, although a second dose was necessary for total removal and prevention of bacterial regrowth. This phenomenon was also observed for other endolysins such as HY-133. In this example, the killing kinetics revealed *in vitro* regrowth of clinical MSSA and MRSA isolates after prolonged incubation (Knaack et al., 2019). This regrowth might occur as a result of endolysin degradation, by saturated binding of the protein to the target bacteria or by regrowth of the surviving bacteria.

Overall, the results presented in this study make LysRODIΔAmi an attractive antimicrobial candidate. More specifically, we consider that this engineered protein has potential for its utilization as a decontaminating agent to be used topically in patients prior to surgery or in farm animals in order to prevent MRSA infections. Further evaluation of a large panel of MSSA, MRSA, and VRSA strains, *in vivo* preclinical analyses and further pairwise comparison with LysRODI will provide insights into the real potential of this lysin.

## Data Availability Statement

The raw data supporting the conclusions of this article will be made available by the authors, without undue reservation.

## Author Contributions

DG performed all the experiments. DG, LR-R, RL, and YB designed the chimeric proteins. LR-R constructed and cloned the proteins. DG, MN, and PR-M designed the experiments related to the HaCaT cell line. PR-M maintained and grew the cell line. DG, MN, and KP designed the *ex vivo* pig skin model. DG and AC obtained the microscopy images. DG, LF, PG, and AR analyzed the data. All authors contributed to writing the manuscript.

## Conflict of Interest

The authors declare that the research was conducted in the absence of any commercial or financial relationships that could be construed as a potential conflict of interest.

## Publisher’s Note

All claims expressed in this article are solely those of the authors and do not necessarily represent those of their affiliated organizations, or those of the publisher, the editors and the reviewers. Any product that may be evaluated in this article, or claim that may be made by its manufacturer, is not guaranteed or endorsed by the publisher.

## Footnotes

1
https://clinicaltrials.gov/ct2/show/NCT01746654

2
https://www.genscript.com/codon-opt.html
