# Supplementary material for: Design and Selection of Engineered Lytic Proteins With Staphylococcus aureus Decolonizing Activity
Source: Front Microbiol. 2021 Sep 14;12:723834. doi: 10.3389/fmicb.2021.723834 (PMC8477017; doi:10.3389/fmicb.2021.723834)
Supplement: Supplementary file 1 [file Data_Sheet_1.docx]

Supplementary Material

Supplementary tables

**Table S1.** MIC values of the parental endolysins and the two most active engineered variants from this work against a collection of staphylococcal strains. MIC is expressed as the mode of three biological replicates. Data concerning the most active proteins against *S. aureus* and their parental endolysins are highlighted in dark grey.

| **Specie** | **Strain** | **MIC (µM)** |  |  |  |  |  |
| --- | --- | --- | --- | --- | --- | --- | --- |
|  |  | **LysRODI^a^** | **LysH5** | **LysA72^a^** | **LysC1C** | **LysRODIΔAmi** | **ClyRODI-H5** |
| *S. aureus* | Sa9 | 0.57 | 3.83 | 1.47 | >42.5 | 0.33 | 1.84 |
|  | 15981 | 1.15 | 3.83 | 1.47 | >42.5 | 0.17 | 1.84 |
|  | 7829 | 1.15 | 3.83 | 1.47 | >42.5 | 1.32 | 1.84 |
|  | 2016-19142-2 | 1.15 | 3.83 | 1.47 | >42.5 | 0.66 | 1.84 |
|  | H1 9/3 | 1.15 | 3.83 | 1.47 | >42.5 | 0.66 | 1.84 |
|  | E10 | 1.15 | 3.83 | 1.47 | >42.5 | 0.08 | 1.84 |
|  | Staph. 10 | 1.15 | 3.83 | 1.47 | >42.5 | 2.64 | 1.84 |
|  | Staph. 11 | 1.15 | 3.83 | 5.88 | >42.5 | 1.32 | 1.84 |
|  | Staph. 15 | 1.15 | 3.83 | 5.88 | >42.5 | 1.32 | 1.84 |
| *S. epidermidis* | F12 | 1.15 | 7.65 | >23.52 | >42.5 | 2.64 | 7.37 |
|  | B | 0.57 | >15.31 | >23.52 | >42.5 | 2.64 | >14.76 |
|  | DG2n | 1.15 | >15.31 | >23.52 | >42.5 | 2.64 | >14.76 |
|  | LO5081 | 1.15 | >15.31 | >23.52 | >42.5 | 1.32 | >14.76 |
|  | LV5RB3 | 2.29 | >15.31 | >23.52 | >42.5 | 2.64 | >14.76 |
| *S. sciuri* | 101 | 4.59 | >15.31 | >23.52 | >42.5 | 2.64 | >14.76 |
| *S. hominis* | ZL31-13 | 1.15 | >15.31 | >23.52 | >42.5 | 1.32 | >14.76 |
| *S. pasteuri* | ZL16-6 | 2.29 | >15.31 | >23.52 | >42.5 | 2.64 | >14.76 |
| *S. xylosus* | ZL61-2 | 1.15 | >15.31 | >23.52 | >42.5 | 1.32 | >14.76 |
| *S. saprophyticus* | ZL112-15 | 0.15 | >15.31 | >23.52 | >42.5 | 0.33 | >14.76 |
| *S. arlattae* | ZL114-5 | 1.14 | >15.31 | >23.52 | >42.5 | 1.32 | >14.76 |
| *S. haemolyticus* | ZL89-3 | 0.57 | 7.65 | 1.47 | >42.5 | 0.66 | 3.68 |
| *S. gallinarum* | ZL90-5 | 1.14 | >15.31 | >23.52 | >42.5 | 1.32 | >14.76 |
| *S. kloosii* | ZL74-2 | 1.14 | >15.31 | >23.52 | >42.5 | 1.32 | >14.76 |

^a^ Data partially published in Gutiérrez et al., 2020.

Supplementary figures

**Figure S1.** Influence of environmental parameters on protein activity: A) temperature, B) pH and C) presence of different ions. Specific lytic activity of the proteins was calculated against *S. aureus* Sa9 in turbidity reduction assays. Proteins were treated at different temperatures (40-90 ºC) for 30 min, or diluted in Britton-Robinson Universal Buffer (pH from 3 to 11) and maintained at room temperature for 1 h, or in 50 mM NaPi buffer supplemented with 1 mM of different ions. The specific lytic activity was calculated by measuring the OD_600nm_ for 30 min at 37ºC and expressed as ΔOD_600_ × min^-1^ × µM^-1^. Bars represent the specific lytic activity relative to the control (value=1) for each protein. Data are the mean ± standard deviation of three biological replicates. Asterisks indicate statistical differences (p<0.05; Student’s t-test) between the specific lytic activity when the protein is subjected to the temperature or pH treatment with the activity observed when the protein is tested at 37ºC in 50 mM NaPi buffer, pH=7.4.

**Figure S2.** Biofilm growth inhibition in the presence of different protein concentrations (up to 2.5 μM). The percentage of biofilm inhibition was calculated by crystal violet staining after growth of biofilms for 24 h. Results are reported as means ± standard deviations of six replicates. The prevention of biofilm formation is expressed as the percentage of the total biomass in each well related to the total biomass in the control wells (without lytic proteins). The dashed line represents the minimum concentration (0.15 µM) needed to obtain maximum biofilm prevention (more than 90%) for the most active proteins (LysRODI and LysRODIΔAmi).

**Figure S3.** Dose-response curve of LysRODIΔAmi against a HaCaT monolayer. Data normalization was performed as follows: first, the normalized-CI at a given time point was calculated by dividing the CI at this point by the CI obtained 10 min after protein addition (normalization time point) for the same well. Afterwards, the normalized-CI obtained for each well was subtracted from the normalized-CI baseline which, in this case, was the control without protein. After this, a dose-response curve was constructed by representing the normalized CI for different concentrations of the protein (represented in the logarithmic scale). Points represent the means ± standard deviations of three replicates. The black line represents the tendency curve (R^2^=0.85). No statistical differences were observed in between the values of the different concentrations according to the ANOVA and SLK post-hoc comparison test (p>0.05).

**Figure S4.** Fluorescence images obtained by CLSM of intact (top) or wounded (bottom) HaCaT cell line after inoculation of *S. aureus* 15981 followed by treatment with 0.7 μM of LysRODIΔAmi. Actin was detected by labelling with Phalloidin-AlexaFluor568 (red) and nuclei were stained with DAPI (blue). *S. aureus* 15981 cells are expressing GFP (green).
